# Long Covid brain fog: a neuroinflammation phenomenon?

**DOI:** 10.1093/oxfimm/iqac007

**Published:** 2022-09-27

**Authors:** Emma Kavanagh

**Affiliations:** Independent Researcher

**Keywords:** neuroinflammation, Dr. Emma Kavanagh, long Covid, inflammatory cytokines, cognition

## Abstract

Neuroinflammation is a process triggered by an attack on the immune system. Activation of microglia in response to an immune system challenge can lead to a significant impact on cognitive processes, such as learning, memory and emotional regulation. Long Covid is an ongoing problem, affecting an estimated 1.3 million people within the UK alone, and one of its more significant, and as yet unexplained, symptoms is brain fog. Here, we discuss the potential role of neuroinflammation in Long Covid cognitive difficulties. Inflammatory cytokines have been found to play a significant role in reductions in LTP and LTD, a reduction in neurogenesis, and in dendritic sprouting. The potential behavioural consequences of such impacts are discussed. It is hoped that this article will allow for greater examination of the effects of inflammatory factors on brain function, most particularly in terms of their role in chronic conditions.

Long Covid is an ongoing problem for large numbers of survivors of SARS-CoV-2 (Severe Acute Respiratory Syndrome Coronavirus 2) infection. One of its more significant, and as yet under explored symptoms is brain fog. Here, we discuss the role of brain fog in Long Covid symptomatology, and consider the impact of this reduction in cognitive function in terms of a Long Covid patient narrative. We then consider the mechanisms likely to contribute to these effects, discussing neuroinflammation and the effects that the activation of microglia can have on cognitive processes, such as learning, memory and emotional regulation [1]. Inflammatory cytokines have been found to play a significant role in reductions in LTP (Long Term Potentiation) and LTD (Long Term Depression) [2], a reduction in neurogenesis [3] and in dendritic sprouting [4]. We consider the role of these systems in the cognitive challenges posed by brain fog, and its effects on learning, memory, emotional regulation and the gender disparity noted in the diagnosis of long Covid. It is hoped that this article will allow for greater examination of the effects of inflammatory factors on brain function, most particularly in terms of their role in chronic conditions.

In early 2020, the world became aware of a new virus. As SARS-CoV-2 (colloquially known as Covid-19) swept the globe, killing millions worldwide, feedback from patient groups began to make clear that the course of this virus was far from simple. Today, long Covid (a patient led term) is a well-recognised potential consequence of SARS-CoV-2 infection. And a significant part of the long Covid experience is brain fog.

I know this. It is now 2 years since my initial SARS-CoV-2 infection, and long Covid has changed my life in so many ways. But perhaps the most distressing is the brain fog. There are days when I cannot think. There are days when things that once came so easily to me, when problems that were eminently solvable are impossible. There are times when it seems that my memory is irrevocably damaged, when I seem to have missed entire conversations that I was undeniably a part of. They call it brain fog. A benign enough sounding word for a condition that can change your life.

While definitions of brain fog are lacking somewhat in their specificity, some have tried to nail it down. Jorgensen (2008) studied the phenomenon within chronic fatigue patients, describing it as consisting of a slowness of thought, trouble focusing and maintaining concentration, issues with forgetfulness and a general haziness in thought processes. Ross *et al*. [6] ranked descriptors of brain fog within Postural Tachycardia Syndrome patients, and found that the main issues reported included forgetfulness, a sense of cloudiness, trouble focusing and communicating.

Davis *et al.* [5] reported that cognitive dysfunction and memory problems were a common feature of long Covid, with approximately 88% of all patients, from all age groups experiencing these issues. Callan *et al.* [7] described the cognitive experience of patients with long Covid as consisting of difficulties in executive function (planning, decision-making, flexibility and working memory), impairments in complex attention (including difficulties in selective, sustained and divided attention as well as reduced processing speed), long-term memory impairments (including problems with free recall, cued recall and procedural memory) and language difficulties (such as problems with word finding and fluency, syntax, reading comprehension and writing). And while it is tempting to think of brain fog as a cognitive issue exclusively, it is also worth bearing in mind the psychosocial effects reported by Callan *et al*. (2022) that stem from these cognitive complaints. Patients reported a deep sense of guilt and shame as they failed to recover sufficiently to return to their previous sense of functioning, and a feeling that others within their social circle did not understand them due to the ‘invisible’ nature of their chronic illness.

It is far from the first time that brain fog has been associated with viral aftermath. Stefano [8] has written a compelling review of historical viral infections that have led to neurological sequelae reminiscent of long Covid, including past flu pandemics and diphtheria. Brain fog has been reported as a feature in the aftermath of Ebola [9] and in chronic fatigue [10] and as a consequence of chemotherapy [11]. From a viral or treatment consequence that has traditionally received, at best, modest levels of attention, long Covid has forced the conversation around brain fog onto centre stage.

## Long Covid brain fog: a patient’s perspective

My initial infection occurred in March 2020, back before there were tests or vaccines, and while it left me sofa-bound for a period of around 6 months, it could arguably be termed mild. I did not need hospitalization and my symptoms, while difficult, remained mostly manageable. It took perhaps 8 weeks for me to start to question, why my recovery was not going as I had anticipated. There were, of course, good days. But those good days would be interlaced with days on which I could barely stand. At the end of 6 months, I still could not walk to the end of my road. Contrast this with the path of infection for my husband and two young sons. For the boys, they complained of headaches, fatigue and, within a matter of days, appeared to have shrugged off the worst effects of it. For my husband, there was the cough, the temperature and the absence of smell. And then the symptoms passed. They all recovered (thankfully).

I, however, did not.

It is fair to say that we know more now than we knew then, that our understanding of how exactly SARS-CoV-2 affects the system has evolved. A recent study in Nature [12] has marked out the changes in brain anatomy following on from a SARS-CoV-2 infection. Research from Libby and Luscher [13] suggests that the initial infection with SARS-CoV-2 triggers an immune reaction within the host. This immune reaction leads to activation of T-cells, neutrophils, macrophages and Mast cells, all of which spread through the body, attacking virus infected cells. This wide spread immune reaction creates a response in the host body, of inflammation that appears to affect multiple systems.

For my children and for my husband, what came next is what we have come to expect as the typical course followed in a SARS-CoV-2 infection. The immune reaction dissipating over time.

The Office for National Statistics estimates that two million people in the UK are suffering from long Covid [14]. A purely clinical definition of the symptoms of long Covid would tell you that they are diffuse and complex, including fatigue, chronic chest and back pain, headaches, post exercise malaise, gastrointestinal issues and cognitive problems.

The experience of it is somewhat more complicated. For many of us, long Covid is a feeling of no longer trusting our own bodies, our own brains. It is knowing that function today does not guarantee function tomorrow. It is intense, bone crushing fatigue, aching limbs. The shaking when your body is no longer under your control. The headache that feels like it cannot possibly remain within your head.

It is that sinking feeling, as you round the top of another hill, feeling the fall coming, the symptoms resurging and praying that you can hold it off, because you have life and responsibilities and a job. It is being told to rest. Even though you have rested, for months and months and months, and that rest has brought no relief. It is being told that recovery comes to those who work for it, that you simply aren’t trying hard enough. It is being told that there is nothing they can do. That no one can help. And anyway, you should feel lucky. There are people a lot worse off than you. And knowing that is true, and hating yourself just a little bit every time you weep for what you have lost.

I am a psychologist and I am an author. And so, in the midst of all the many symptoms and consequences of long Covid, it is perhaps brain fog that has proven the most damaging to me. I need to think, I need to be able to form sentences, to express my thoughts. And yet, when the brain fog comes, as it often does when I am tired, or when I have pushed my failing body too hard, thoughts become impossible. It is well named, because the feeling is one of a haze being lowered across your thoughts, everything becoming murky and indistinct. To say I have trouble remembering would be a colossal understatement. My memory appears to be the part of my cognition that has suffered the most. Things that I do routinely now require post-it notes, iPhone reminders. Because otherwise, I will forget to take the washing out of the machine, or will not know what to order at our favourite takeaway, even though I have been placing the same order for years now. I forget conversations I have had. And memory prompts are no longer sufficient to bring them back to me. Rather it is as if they have been wiped clean from my mind, as if, as far as my brain is concerned, they simply never happened.

Attentional processes have been compromised. I struggle to sustain attention, to keep my focus where it needs to be. I find myself so easily overwhelmed now, as the amount of sensory information I am used to receiving (the type you get when you live with a husband and two young kids and two dogs and a cat who thinks he’s a dog) is now almost impossible to process. There are times when I am reduced to tears by the excess of sensory input. Which brings me to emotional regulation—I cry a lot more than I used to.

For the first year of long Covid, the brain fog symptoms I experienced were, for me, catastrophic. I could not think sufficient to watch a TV show, let alone enough to write the novel I was mid-way through. It is hard to describe the feeling of your entire cognitive apparatus grinding to a halt, particular when your whole career depends on it. For a year, I could not even think enough to worry about the position I was in, about the fact that I was a self-employed author who could not write.

Things began to shift, painfully slowly, at the end of that first year. My ability to think creatively began to come back online again. I was able to concentrate, albeit for short bursts of time. And slowly, the brain fog began to recede, back to where it is now. Two years on, there is no doubt that it has improved. I can think, I can create. And yet, the consequences of what has happened have been stark. For a year I did not work. That means that books that should have been delivered have not been, and so payments that should have come in never were. Instinctively, I try to push myself, to get all the work that I failed to do then done now. But this strategy invariably fails, as the limits imposed on my body and brain by long Covid kick in. Even now, I have a couple of hours, perhaps half a day if I’m lucky, where I can work intensively before the crash comes, and the fatigue closes in and the fog lowers. Sometimes I am able to push through, to force myself to get the job done, to concentrate for far longer than I know my body is willing to endure. The consequences then though are severe, almost invariably leading to not only intense brain fog, leaving me unable to do more than sit and stare, but also to the full gamut of long Covid symptoms that I have come to know over the past 2 years. The chest pain, the headaches, the crippling fatigue, the shakes and the muscle pain. They are never too far away from me now.

The limits to my brain function thanks to long Covid have changed my life entirely. I cannot trust myself to know the right words to say, to remember what is important. And it is hard not to feel like a failure, when I realise that I have once again forgotten something I had sworn I would remember. Because of all the time I lost, I need to push myself now, to get my writing done, to work harder to back fill that financial hole. But I can’t, because my body will not let me. And so I have replaced hard work with harder worry.

## Long Covid brain fog: a neuroinflammation phenomenon?

This is not the first time cognitive factors have been related to chronic conditions—for example, in the long-term sequelae of Ebola [9] and in chronic fatigue [10], among others. These cognitive factors are, however, often treated as a side note, a brain anomaly in a body focused condition. And yet, for many, the consequences of such impaired neurological functioning are catastrophic. Many of those struggling with long covid are faced with the ongoing challenges of adapting their work/life circumstances to the demands of this chronic ailment. Such adaptations demand no small degree of cognitive flexibility and problem solving, as energy limits compromise their ability to return to their normal level of functioning. To treat this symptom as an aside is, I feel, to greatly undervalue its impact on those affected by it.

Efforts to explain brain fog have been largely clouded by this designation of the problem as a side effect. However, a number of researchers [15, 16] have suggested a role for chronic inflammation, triggered by the immune response. Darley *et al.* [17] compared recovered covid patients with those symptomatic for long Covid, and found that, in the latter group, evidence of diffuse inflammatory cytokines could be found. Their study concluded that this immune-related inflammation may be the driving force behind long Covid symptoms. Further support from these findings has come from the cerebrospinal study conducted by Jarius *et al.* [18], which reported strong activation of inflammatory pathways for an extended period following Covid infection.

We are all familiar with the feeling of being ill. That sense of fatigue, that one’s thinking has become muddied, difficulty staying on task. ‘Feeling ill’ is, rather unsurprisingly, the behavioural outcome of our body’s immune system activation. When the body reacts to an immune system challenge, inflammation is seen throughout the central nervous system, a process referred to as neuroinflammation.

When the body comes under attack from possible sources of infection, the glial cells act as the first responders. Abundant and spread throughout the central nervous system, glial cells assess their environment, keeping an eye on our physiological functioning and springing into action when a threat is detected. Including astrocytes, microglia and oligodendrocytes (to name but a few), these cells are critical in the way in which our bodies react to an immune system challenge.

One of the principle features associated with the defence of the nervous system are microglia. During development, microglia are responsible for the coordination of synaptic pruning, allowing the brain to eliminate weak synapses, leading to the strengthening of those that are more functional. As we age, microglia also play a roll in synaptic clearance. Microglia are responsible for regulating memory, by the release of brain-derived neurotrophic factor (BDNF) and the promotion of synapse formation. They are also charged with the constant surveillance of the nervous system, on the lookout for potential immune system threats.

When such a threat is detected, the microglia are activated, making them the first line of defence to immune system challenges [19]. This microglia activation initiates the release of cytokines and chemokine, thus creating an inflammatory signalling process. This activation and its resultant inflammation are usually fleeting, lasting long enough to deal with the immune system challenge. However, when the activation of microglia become impaired, it can lead to a prolonged expression of inflammatory cytokines [20].

According to a recent study by Probstel and Schirmer [21], sustained activation of microglia and astrocytes is likely to play a pivotal role in the chronic neuroinflammation following on from SARS-CoV-2 infection. Inflammatory cytokines are critically involved in the process of learning and memory [2]. Their presence affects long-term potentiation (the sensitization of neurons due repeated synaptic excitation), thus leading to more efficient signal transfer along the potentiated neurons. This allows us to build up connections, to absorb new information. Cytokines are also known to affect long-term depression, the process by which neurons become desensitized. As these functions play an integral role in synaptic plasticity, the ability of the brain to reshape its neural circuits, the individual’s ability to show cognitive flexibility is critically dependent on the body’s immune reactivity.

The effects of inflammatory cytokines on the brain can be described by using an inverted U shape. A certain level is critical for effective neural functioning. Beyond this level, however, problems can arise. This means that either their inhibition or excess can create a change in cognitive function [22].

When considering long Covid and its potential relationship to chronic inflammation, its important we consider what happens in the brain when a long-term over-expression of cytokines becomes the norm. Much research in this area has concentrated on the hippocampus, due to its significant role in learning and memory, which means that we know more about how cytokines operate here than in some other areas of the brain. In 2009, McAfooze and Baune reported that high concentrations of the cytokine interleukin-1β (IL-1β) was associated with a reduction in long-term potentiation in the hippocampus. Other studies [23] have shown that over expression of IL-6 led to reduced neurogenesis (the formation of new neurons) in the hippocampal dentate gyrus, a finding further supported by Wu *et al.* [24]. Gonzalez *et al.* [4] found that stimulation of the immune system led to a decrease in BDNF expression. They proposed that it was this decrease that served to impact synaptic plasticity in the hippocampus and compromise the brain’s ability to engage in neurogenesis, LTP and dendritic sprouting.

In practical terms, what this means for me and for people like me is that we will potentially see problems in learning and in memory, that it will become harder for us to absorb new information, to build new neural connections.

Which begs the question, can long Covid brain fog be explained by the effects of neuroinflammation on the hippocampus? Likely, this theory is insufficient to explain all of the cognitive effects of long Covid.

We know that excess cytokine levels affect functioning of prefrontal and limbic structures [25] and that IL-6 and TNF (Tumor Necrosis Factor) levels are correlated w/activity in the insula and the ACC (Anterior Cingulate Cortex) [26], thus suggesting that, where we conclude that brain fog was indeed a function of neuroinflammation, we would then have to go further, in order to establish what impacts it was having on these other areas of the brain and how they, in turn, might be affecting behaviour.

It is worth noting that the neuroinflammation literature raises other issues that may be playing out in long Covid. Cytokine activation is known to influence dopamine levels [27], a process which leads to the inhibition of reward motivation and anhedonia. Likely this will have effects on an individual’s capacity to perform their typical roles, be they professional or personal. It should also be noted that although much of the human-based research has focused on inflammation associated with pathological conditions, Vintimilla *et al.* [28] found a relationship between C-reactive protein and verbal fluency and executive function in healthy individuals. Which would therefore beg the question, how much inflammation would be required before an effect was perceived by the individual?

It would be remiss of me to finish this article without reference to the impact of neuroinflammation on mood. It has been repeatedly noted that there is a greater vulnerability towards long Covid in those suffering from comorbidity disorders such as depression and anxiety [29]. A finding that has led to the painfully inevitable ‘All in the head’ chorus. Well, yes … but not in the way that such conclusions suggest. Evidence has repeatedly shown a link between inflammation and depression [30]. Dantzer *et al*. [31] reported that the systemic injection of pro-inflammatory cytokines induces depression type behaviours, while Guo *et al.* [32] specified the role of cytokines IL-1, IL-6, TNFα and INF
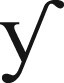
It has been theorized [33] that these cytokines directly affect those regions of the brain involved in regulation of emotion. Perhaps then, the links between anxiety, depression and long Covid are more reflective of these conditions sharing a neuroinflammatory link.

It should further be noted that the greater proportion of women diagnosed with long Covid Lindahl et al. [35] could quite logically be explained by this same neuroinflammation process. Klein and Flanagan [34] found that women tend to react to immune system threat with a stronger inflammatory response. They theorize that this increased response has served us well in evolutionary terms, providing girls and women with a more robust response to clinical acute infections. They suggest that there is, however, a down side to this increased reactivity—that when inflammation fails to resolve and becomes chronic, it can create a greater vulnerability to chronic conditions such as long Covid.

We do not have the answers yet. But over the past 2 years, we have found clues, indications as to what exactly may be driving this swathe of long Covid suffering. While I hope that the above might provide some insight into the role that neuroinflammation can play, I am under no illusions that it is enough. The evidence surrounding neuroinflammation appears to go no small way towards explaining so many of the cognitive symptoms of long covid, and yet further research is needed. How much inflammation is sufficient to cause these symptoms? Which cytokines are most relevant in any inflammation factors associated with long Covid? And, most importantly, what can we offer in terms of treatment?

And finally, I hope that this article might serve as a plea to those within the research community. We need help. The world is different now, and so we need to do things differently. We need to communicate, to share the ideas we have with the communities around us, the scientific, the experiential, the medical and the psychological. We need to break through the bottleneck that exists between scientific research and the world beyond, so that our findings, our theories, our data, can be shared with those who need it most. It is undeniable that the people who have the most information about this condition are those who have lived with it, for months, for years. These same people who drove the recognition of the condition itself will be at the vanguard of reaching an understanding of it. They will be, because they have to be. Because for them, for me, we need this if we are to survive. Their experiences, their understandings, what they can bring to the knowledge we have about this condition, is critical and unparalleled in any purely academic setting. We need to harness this, to expand our definition of where valuable information comes from. We need to involve them in this quest for answers. Not merely because of what they can contribute, but also because of what this quest for answers can contribute to them. Hope. Because we need to have hope that answers will come, even if we have to find them for ourselves.
